# Pathways for pragmatic decolonisation in research

**DOI:** 10.12688/openreseurope.19943.1

**Published:** 2025-04-17

**Authors:** Monique Kwachou

**Affiliations:** 1 University of Bristol Faculty of Arts, Bristol, England, UK

**Keywords:** Decolonising, knowledge production, Epistemic decentralisation, Africa

## Abstract

**Background:**

For too long, research on African peoples, histories, and ideas has been shaped by institutions and frameworks rooted in former colonial metropoles. This has sustained epistemic hierarchies that privilege Western paradigms while marginalising African knowledge systems. While there is increasing consensus on the need to decolonise research, less attention has been paid to how this can be achieved in practical terms. This paper argues that decentralisation—a concept familiar in governance—offers a useful metaphor and framework for rethinking how power over knowledge production can be redistributed to African scholars, institutions, and communities.

**Methods:**

With this paper, the author adopts a conceptual-empirical approach grounded in personal research experiences within Cameroonian higher education and supported by a review of scholarly efforts by African researchers engaging with decolonial paradigms. Reflexive narrative inquiry is used to interrogate how decision-making, methodological choices, and epistemic validation processes unfold in research spaces. The paper reinterprets decentralisation to develop a framework for epistemic redistribution in knowledge production.

**Results:**

Building on the idea that decolonisation entails decentralising epistemic power, the paper identifies one foundational starting point and three interconnected pathways for action. Theoretical pathways reclaim African epistemologies as valid and generative, disrupting Eurocentric dominance. Methodological pathways advance participatory, Afrocentric approaches grounded in lived experience and relational ethics. Administrative pathways call for institutional reforms that empower African scholars and communities in shaping research agendas, resource flows, and dissemination. Collectively, these pathways outline intentional shifts in authority over theory, method, and governance that operationalise decolonisation in knowledge production.

**Conclusions:**

By re-framing decolonisation as decentralisation, this paper provides an accessible and actionable model for transforming knowledge production in African contexts. It contributes to bridging the theory-practice gap in decolonial discourse, offering concrete strategies to recentre African thought, amplify historically marginalised voices, and cultivate epistemic justice within and beyond the academy.

## Plain language summary

For long, research about African people, history, and ideas has been controlled by institutions of former colonial metropoles that now make up the historically privileged Global North. This has led to a situation where Western thinking is seen as the "right" way to do research, while African knowledge systems and perspectives are often ignored or undervalued. Many scholars have called for change, arguing that knowledge production worldwide should be more inclusive and reflect the voices of African (and other indigenous) communities. However, while there is a lot of discussion on why research should be decolonised, there is less clarity on how to actually make it happen practically.

This paper suggests that one way to understand decolonising research is through the idea of decentralisation—a concept already familiar to many people globally, as so many governments use it. Decentralisation means shifting decision-making power from a central authority to local communities, giving them more control. Here, the author argues that we can apply the same idea to knowledge production to make decolonisation easier to understand and implement.

Just as decentralisation shifts decision-making from central authorities to local communities, decolonising research involves re-distributing power over knowledge production to ensure African scholars, institutions, and their communities have greater influence in determining research priorities, methodologies, and dissemination practices.

The author then states that when we see decolonising as the decentralisation of power over knowledge production, it reveals that there are three broad ways- following a starting point- by which those who want to decolonise research can go about it. The paper presents details on the identified starting point and the pathways using examples from empirical research by the author and other scholars.

## Introduction

Over half a century since most of the world's colonised nations ostensibly achieved political sovereignty, the concept of decolonisation remains topical. This enduring relevance stems not only from the fact that some territories remain colonised to this day ^1^ but also from the legacies of colonial rule that continue to shape global structures, from governance to socialisation and, most relevant to this paper, knowledge production ( Aboderin *et al.*, 2023; Ndlovu-Gatsheni, 2013a). Hence, contemporary calls to decolonise go beyond the historical acts of reclaiming land and political power; they extend to dismantling the ideological, epistemic, socio-cultural, and economic systems that continue to uphold the benefits of colonialism for former oppressors and sustain the marginalising of historically oppressed groups.

With persistent and mounting calls to decolonise, the discourse of decolonisation has expanded across disciplines and spaces, with calls to "decolonise" everything ^2^ from museums to therapy, from humanitarian aid and religious institutions. While this proliferation underscores the concept's broad applicability, it has also risked conceptual dilution. The more widely the term is applied, the more difficult it becomes to discern its core tenets, leading to ambiguity about what decolonisation entails in practice. Scholars increasingly claim that the decolonisation movement has 'lost its way' and become co-opted to a superficial end ( Adébísí, 2019; Táíwò, 2022). A notable critique of this phenomenon comes from Tuck and Yang (2012), who argue that decolonisation is often treated as a metaphor for social justice or personal enlightenment- particularly by educators influenced by Paulo Freire. They contrast this with Frantz Fanon's framing of decolonisation as a concrete, material process addressing land dispossession, racial hierarchies, and settler sovereignty ( Tuck & Yang, 2012, p.19–20).

While I find such critiques insightful and the warnings of such scholars worth heeding, I feel some of the critiques ( Táíwò, 2022; Tuck & Yang, 2012) often apportion blame for the issue at hand wrongly; that is to say, they are not baseless critiques, but they have the wrong/insufficient informational bases. Critiques such as Tuck & Yangs (2012) and Táíwò (2022) suggest that the concept of 'decolonising' be straight-jacketed. In their critique of the broader application of the concept of decolonising, Tuck and Yang (2012) seemingly refuse to acknowledge the distinction between colonialism—the historical act of domination—and coloniality, the enduring structures of power that persist long after formal colonial rule ended ( Maldonado-Torres, 2007; Mignolo, 2013; Ndlovu-Gatsheni, 2013b; Quijano, 2000). Likewise, when Tuck and Yang (2012) dismiss certain calls to "decolonise" as metaphorical, they risk suggesting that countering coloniality is a less critical 'social justice issue' compared to countering colonialism. While colonialism—the tangible act of dispossession—remains a crucial issue, coloniality's more subtle, pervasive influence on the ways of life via knowledge systems is no less significant. Efforts to decolonise knowledge production are means by which we reclaim intellectual sovereignty in ways that challenge the global structures that sustain epistemic inequality, amongst other disparities. Coloniality is not simply a residue of colonialism; it actively sustains global inequalities through knowledge production, economic systems, and cultural hierarchies. Thus, while efforts to decolonise research may not always directly address land dispossession, they remain critical for reclaiming power for the historically oppressed by dismantling the epistemic foundations of coloniality that continue to shape the world. Both are valid and necessary as they address different dimensions of systemic oppression.

Táíwò (2022), on his part, recognises the differentiation between coloniality and colonialism but insists that the word 'decolonising' outside of independence struggles is wrong and the expansion of the concept into domains of cultural studies that has wrought "confusion and obscurantism if not outright distortion and falsification" (p. 20). Personally, I opine that 'decolonising', like countless other words in our vocabularies, can refer to more than one thing and can be used in more than one way; 'decolonising' is clearly an active word that denotes a process in ways decoloniality would not when paired with another word. ^3^ Hence, using 'decolonising knowledge production' to refer to the action born of the school of thought of decoloniality ought not to be blamed for 'distortion' or 'falsification'. I would argue that behind the uncritical deployment of the concept of decolonising ( Adébísí, 2019) lies the central problem of scholarly vagueness and the failure of thinkers and scholars of decolonising to come up with shared objective standards for truth ( Adébísí, 2019) – of what pursuits like 'decolonising knowledge production' might look like and how one can go about it. It is this lack of clarity in theorising and applicability of decolonisation with respect to knowledge production (among other things) that often turns it into a metaphor—this is what I set out to address in this paper.

It is necessary to clearly state that although I have begun with critiquing some critiques of contemporary decolonising discourse, my aim with this paper is not to address highbrow scholarship, nor are such thinkers my audience. The reason I began with this is that I fear such takes and ideas that we need to "rid ourselves of decolonisation" ( Táíwò, 2022) or pause the discourse for fear of its continued co-opting, risk "throwing the baby out with the bathwater" and derailing the movement before it actually has significant impact where it is most- outside of theoretical discourse.

You see, the much-contested calls to "decolonise knowledge" are not new; they trace back to the African nationalist struggles of the 1950s and have been sustained by decades of critical scholarship challenging the dominance of Western epistemological frameworks ( Crawford *et al.*, 2021). Yet, despite the depth of this intellectual tradition, the practical implementation of decolonial thought in and out of [specifically African] academic institutions remains limited. Reflecting on my own academic journey as a Cameroonian scholar, I only encountered the idea of decolonising knowledge production in 2015 while conducting my master's research at University College London (UCL) and engaging with other African students and scholars of African studies far away from home- despite having completed undergraduate studies in Sociology and Gender Studies—fields deeply engaged in questions of power, identity, and social structures—at the University of Buea in Cameroon. This belated exposure illustrates the extent to which decolonial discourse remains largely disconnected from African higher education curricula and, consequently, from the experiences of novice African scholars and broader society outside academia.

My subsequent experience as a lecturer at the University of Buea reinforced this observation. Returning to teach Gender Studies, I discovered that undergraduate students were largely unfamiliar with key African-feminist scholarship and rhetoric, and when I raised this issue with colleagues, the standard justifications were that such material was too complex for undergraduates, that students would have time to engage with it independently at the master's level (see Kwachou, 2023), or that they would eventually develop the ability to decode the highly academic and abstract language in which much decolonial scholarship is written. Most recently, in my ongoing work as lead of the Cameroonian case study of the European Research Council (ERC)-funded *Creative Lives of African Universities* project (to be elaborated upon later), I found that the student co-researchers—ranging from undergraduates to doctoral candidates—were either as unfamiliar with decolonial research discourse as I had been when I first encountered it abroad or grasped its importance but struggle to envision what decolonising knowledge production entailed in practice. This suggests that despite decades of scholarly engagement with decolonising, understanding and implementation of these perspectives into mainstream (in and out of education) remains inaccessible and beyond reach. Hence, with this paper, I propose that rather than "getting rid of decolonising" knowledge production and further critiquing where the movement has derailed, we put more effort into demystifying it.

With this paper, I attempt to do just that: to provide clarity and outline pragmatic guidance on decolonising knowledge production, particularly for novice students across various disciplines, in hopes of contributing to ongoing efforts to move decolonisation beyond rhetorical engagement and towards practical transformation within academic institutions. To this end, the paper makes two key contributions.

First, I propose understanding decolonising research through the lens of decentralisation. Unlike decolonisation, decentralisation ^4^ is widely recognised, as nearly all countries employ it to an extent in governance ( Rugeiyamu & Msendo, 2025). Generally, decentralisation refers to the redistribution of decision-making power from a central authority to local entities to enhance efficiency and responsiveness ( Crawford & Hartman, 2008; Yuliani, 2004). Viewing decolonising knowledge production as the decentralisation of epistemic power makes it more accessible and actionable, especially for emerging scholars.

Secondly, I aim to demonstrate how viewing decolonisation as epistemic decentralisation enables scholars to recognise the diversity of approaches and varying degrees of decolonising knowledge production. Existing research typically defines decolonising research as (1) employing a method deemed "decolonial," (2) adopting ethical protocols that respect Indigenous subjects, or (3) citing Indigenous theories. Few studies integrate all three. However, framing decolonisation as the decentralisation of epistemic power moves scholars beyond these limited approaches by highlighting that any shift in epistemic power—regardless of scale—represents a degree of decolonisation. Embracing this perspective allows for a broader, more dynamic understanding of how research can be decolonised. Drawing from my research in Cameroonian higher education and examples from other African scholars, I identify three broad pathways (and an initial starting point) that encapsulate [common] approaches to decolonising knowledge production and demonstrate how each pathway reflects a way of decentralising epistemic power, with varying degrees and forms.

The paper is structured into five sections. Following this introduction, the second section reviews scholarship on decolonising research and introduces the correlation between decolonisation and decentralisation. The third section examines relevant studies—both my own and those of other scholars—that illustrate collaborative, pragmatic approaches. The fourth section outlines the three broad pathways I identify in decentralising epistemic power, beginning with the critical "starting line" necessary before engaging in any approach. The conclusion reiterates the central argument of the paper.

## A case for viewing decolonisation as epistemic decentralisation

In writing this paper, I am guided by the fundamental question: Where would one begin if one were to teach an introductory course on decolonising knowledge production to novice researchers of diverse fields in and out of academia? What would enable them to better understand and pursue decolonising in research? I suppose one would begin as most fumbling novice scholars do, as I myself once did, by a review of scholarship on decolonising research. Any such review would reveal the resurfacing of some key scholars whose work on the subject is widely referenced, shared perspectives, recurring themes, and persistent debates and pragmatic issues across decolonising discourse.

### A review of decolonising knowledge production discourse

Seminal thinkers who have shaped this area of scholarship by demonstrating how colonial structures continue to influence our understanding of what constitutes valid knowledge and who gets to produce it include Frantz Fanon, Ngũgĩ wa Thiong'o, Paulin Hountondji, Linda Tuhiwai Smith, Nelson Maldonado-Torres, Walter Mignolo, Boaventura de Sousa Santos, and more recently Bagele Chilisa, Marie Battiste, Achille Mbembe and Sabelo Ndlovu-Gatsheni.

Fanon ( 1952; 1968), regarded as a visionary in this field, critiqued colonialism's psychological, cultural, and structural impacts, emphasising that decolonisation involves not only dismantling colonial structures but also reclaiming intellectual and cultural autonomy. Building on Fanon, thinkers like Ngugi (1986) highlight the centrality of language in reclaiming Indigenous epistemologies, while Smith (1999) critiques Western research's extractive tendencies and advocates for methodologies rooted in Indigenous worldviews that prioritise community participation and benefit. Quijano ( 2000; 2024), as earlier mentioned, made a groundbreaking contribution to decolonial scholarship with his conceptualisation of the coloniality of power, a framework that reveals the enduring influence of colonial structures within contemporary institutions and emphasises the urgency of reclaiming epistemological autonomy for the colonised. Building on this foundation, scholars such as Maldonado-Torres (2016) and Mignolo ( 2012; 2013) have expanded the discourse on coloniality, each offering critical perspectives on its manifestations and resistance. Mignolo (2009), for example, advances the notion of 'epistemic disobedience', urging scholars to reject Eurocentric paradigms and embrace a pluriversal approach to knowledge. In a complementary vein, de Sousa Santos ( 2015; 2017) introduces the concept of 'epistemicide', describing the systematic erasure of non-Western ways of knowing. He advocates for an 'ecology of knowledge', a framework that upholds the coexistence of diverse epistemological traditions without imposing a hierarchical structure.

Hountondji's ( 1990; 1997; 2006; 2009) work significantly advanced the African decolonising knowledge discourse; he critiqued the romanticising indigenous knowledge without critical engagement, saying the mere descriptive acknowledgement of African philosophies and the framing of them as 'indigenous' renders African knowledge and ways of knowing as static and pre-modern, he advocated instead for the use of 'endogeneity', which focuses on where knowledge originated from (within African contexts) rather than whether it was there before imperialism. For Hountondji, decolonisation does not mean a rejection of universality but rather dismantling epistemic hierarchies and engaging often marginalised perspectives to create conditions for more diverse, egalitarian and genuine universal discourse ( Negedu, 2024). His theorising positioned decolonisation as an opportunity for global epistemic exchange that fosters intellectual parity ( Hountondji, 2006).

The works of other African seminal scholars of decolonising knowledge production discourse (see Ndlovu-Gatsheni, 2013b; Ndlovu-Gatsheni, 2018) and Mbembe (2019) reveal recurring themes: the persistence of coloniality sustained through epistemic structures and traditions, urging critical reflection to transform entrenched hierarchies, challenging Eurocentric dominance, re-centring and validating the oft-invalidated indigenous knowledge, and addressing power imbalances in research. However, barely any seminal scholars noted above clearly stipulate how decolonising knowledge production can be practically pursued or what actionable steps young scholars engaging in research should take—besides questioning and/or rejecting Eurocentric practices (see Woldegiorgis, 2025).

In this respect, Bagele Chilisa is an exception. Chilisa's seminal work *Indigenous Research Methodologies* ( 2012) stands out for clearly presenting the postcolonial Indigenous research paradigm as emanating from the lived experiences, values, and history of those communities marginalised by Euro-Western approaches. Chilisa not only explains why decolonising research matters but provides practical guidelines for doing so through relational Indigenous methodologies that foster collaborative, community-inclusive research that revitalises lost identities and value systems. Chilisa offers practical methodologies that collaborate with communities, restore lost value systems, and provide strategies for researchers. She further introduces an "Indigenous research continuum scale", allowing both Indigenous and non-indigenous researchers to evaluate their decolonising efforts, ranging from "least indigenised" to "geocentric or third space methodologies" ( Chilisa, 2012, p.28–31).

***The challenges of decolonising knowledge production discourse.*** While the above presentation of discourse on decolonising knowledge production establishes that foundational and contemporary scholars have contributed significantly to theorising decolonisation, this area of scholarship remains highly contested, fraught with ideological tensions, practical constraints and little consensus on what it entails or how it should be pursued in practice. As a result, decolonising knowledge production risks remaining an ambitious yet fragmented ideal rather than a structured, actionable movement in and out of academia.

Woldegiorgis (2025) notes that one of the fundamental challenges is a lack of conceptual clarity. Although the decolonisation movement broadly agrees on dismantling colonial legacies in curricula, institutional structures, and knowledge production, it lacks a unified definition of decolonising knowledge. Promoters of decolonising knowledge production advocate for centring African epistemologies, histories, and intellectual traditions, yet they differ on the specifics of how this should be achieved and the extent to which is can, whether decolonisation requires radical rupture or reformist inclusion. Fanon's call for a complete break from colonial structures remains supported by many in this area of scholarship, yet the feasibility of such a rupture in an era of global intellectual interconnectedness is just as widely questioned. While some argue that severing ties with colonial epistemologies is neither practical nor desirable, others critique reformist measures—such as diversifying reading lists or renaming institutions—as superficial solutions that fail to dismantle underlying power structures.

Moreover, in seeking to validate indigenous and subaltern epistemologies, decolonial thinkers have unintentionally reinforced a rigid binary between "Western" and "African" knowledge systems. This framing oversimplifies the diversity of intellectual traditions across cultures and risks reducing decolonisation to solely a counter-hegemonic struggle rather than a nuanced reimagining of knowledge production. With such a binary, widely accepted definitions of decolonising research 'the inclusion and equal valuation of Indigenous ways of knowing' (see Held, 2019) or Chilisa's ( 2012, p. 11) description of it as "centring the concerns and worldviews of the colonised other," unintentionally risk encouraging the idea that decolonising is about creating an indigenous equivalent of western ways of knowing and playing catch up or worse fostering "add-and-stir" approaches that integrate indigenous knowledge without transforming the structures of power that determine whose knowledge holds authority.

Furthermore, by positioning decolonisation primarily as a binary struggle between Western and non-Western epistemologies, scholars risk overlooking the epistemic hierarchies that also exist within indigenous spaces. An effective decolonial framework must not only critique colonial exclusions but also account for how marginalised groups themselves may reproduce epistemic exclusions.

Beyond theoretical debates, the practical implementation of decolonising knowledge production presents its own set of challenges. Many foundational scholars provide critical analysis but stop short of offering clear strategies for applying decolonial principles in research. Ngugi wa Thiong'o's advocacy for linguistic decolonisation, for example, remains influential, but his call for a return to indigenous languages in education does not fully address the potential exclusions this could create in multilingual African societies. Even where decolonial scholars offer pragmatic steps for decolonising- such as Smith (1999) and Chilisa (2012)- they focus on specific dimensions of knowledge production, like ethics and methodologies. A holistic framework is needed to address what decolonising would look like throughout the entire research process—from conceptualisation to dissemination, epistemic validation, and structural reform ( Woldegiorgis, 2025).

As though in response to the absence of such a comprehensive framework, there has been a proliferation of initiatives aimed at promoting equity in research partnerships and the knowledge economy. Over the years, numerous guidelines and toolkits have been developed to address epistemic inequities, including the UKRI-led framework on equitable partnerships, the Swiss Academies of Sciences' guide for transboundary research, the global Declaration on Knowledge Equity, the ERC's own Global Code of Conduct for Equitable Research Partnerships, and the Association of Commonwealth Universities' Equitable Research Partnerships Toolkit. In Africa, the African Charter for Transformative Research Collaborations has emerged as a leading institutional commitment to decolonisation.

While these initiatives signal progress in bridging the gap between decolonising research theory and praxis, they remain limited in scope. Most focus on Global North-South partnerships as the primary site for decolonisation, overlooking the ways coloniality is reproduced within African institutions, South-South collaborations, and the work of individual scholars. Additionally, while these frameworks provide general principles for equitable research, they do not target the average thinker nor even early-career researchers; hence, they are actionable steps for those who may struggle to integrate decolonial methodologies into individual research work.

For instance, the African Charter for Transformative Research Collaborations (2023) identifies six layers of power asymmetries in global knowledge production and outlines ten principles for addressing them. While these principles provide a valuable foundation, they do not translate easily into day-to-day research practices for individual scholars. I must note that an exceptional example of progress in this regard is the Sage Research Methods guide entitled *How to Share Power in Research: An Eight-Step Model Toward Decolonizing Research Ethics and Building More Equitable Research Partnerships* ( Cascant *et al.*, 2024). Unlike many other resources, it offers tangible steps that individual researchers can follow to address power asymmetries at different phases of research explicitly. Still, the diversity of decolonising knowledge production needs makes the eight-step model a bit restrictive and suggests that decolonising knowledge production is done only upon checking off that list, which is problematic.

Another major shortcoming of decolonial discourse and its associated toolkits is the inadequate confrontation of financial and economic structures that sustain coloniality. While decolonial scholars (see Crawford *et al.*, 2021; Ndlovu-Gatsheni, 2018) and documents like the African Charter for Transformative Research Collaborations (2023) acknowledge financial disparities as a key factor in the continued marginalisation of Global South scholarship and make calls for more 'equitable financing', their framings often fail to adequately confront the fact that these disparities were deliberately engineered to serve specific economic and political interests and answer the fundamental question: why would those who benefit from epistemic dominance willingly relinquish their privilege? Without a framing that adequately addresses this question, calls for decolonising risk presenting as appeals to Global North's scholarly 'conscience' or 'aid' and thus depend on individual and institutional whims. As is, the presentation of decolonising research partnerships as necessary for a thriving "global knowledge economy" is misleading as it obscures the reality that the actual currency of knowledge production is not merely information but the fiscal resources—dollars, pounds, and euros—required to fund research and intellectual work. African universities, heavily reliant on Northern funding, global rankings, and external grants, find their research priorities shaped by financial imperatives that often run counter to decolonial aspirations. Institutions with greater financial resources are better positioned to meet dominant academic benchmarks, yet this pursuit of global recognition frequently comes at the expense of genuinely transformative decolonial projects ( Oelofsen, 2024).

It is in consideration of the valuable contributions of the plethora of scholars to decolonising knowledge production, as well as the challenges that arise within the discourse and applications of it, that I submit the need for and benefits of framing decolonising knowledge production as the decentralising of epistemic power. If a new scholar were to enter this field, they would encounter a landscape of powerful theoretical critiques and scattered practical examples that address facets of decolonisation without providing a holistic perspective on it. The lack of an encompassing framing of what decolonising knowledge entails and lack of pragmatic illustration of how it can be pursued and what it would look like, be that at the individual or institutional level, is, I believe- what renders it inaccessible and much challenged today, and from recent writing of scholars like ( Woldegiorgis, 2025), others agree with me.

For this reason, I argue that foundational scholars' breadth of theorisation and conceptualisation of decolonising research and the attempts and development praxis via declarations and toolkits need to be ordered together and presented as pieces of one puzzle rather than contradicting views. Woldegiorgis (2025) notes that without a unified framing to consolidate the various dimensions of decolonising knowledge production, this movement risks remaining aspirational rather than transformative. Yet one uncontested idea remains central to decolonial thought: knowledge is power, but how it is produced and whose knowledge is valued has created and or sustained disparities in power globally. Hence, knowledge production is inherently political. This single tenet holds across all arguments on decoloniality and decolonising knowledge production. Irrespective of how scholars differ on how to reimagine knowledge systems, it is clear that it is the efforts to re-distribute power, the power of knowledge, and the power imbalances that coloniality has sustained that are being pursued when decolonising. Thus, I propose that we view decolonisation as fundamentally about the decentralisation of epistemic power. This perspective captures the essence of decolonisation as described by scholars across different contexts, and framing decolonisation as the decentralisation of epistemic power provides a unifying lens that encompasses the various approaches and makes it easier to demonstrate the different contributions made by scholars.

### Framing Decolonisation as Epistemic Decentralisation

My drawing of a parallel between decolonisation and decentralisation is not altogether novel; scholars have previously postulated their interconnectedness. Mignolo (2000), in *Local Histories/Global Designs: Coloniality, Subaltern Knowledges*, for instance, highlights the complementarity of these concepts where he frames the liberation of colonised peoples as contingent upon challenging colonial epistemic dominance and the dismantling of the authority and centrality of Eurocentric epistemologies to re-centre marginalised voices and perspectives as central to decolonising (pp. 20–21). More recently, Medina ( 2024, p.1) asserts that "a process of decentring is currently being observed, evidenced in proclamations such as decolonising the university and the curriculum, provincialising disciplines, opening up the sciences, reconfiguring canons". Hence, I build on existing scholarship as I propose that the concept of decentralisation be leveraged to articulate the theory and advance the practice of decolonisation in knowledge production.

The concept of decentralisation traverses multiple contexts, fields and disciplines. From finance and management to network communication to education and political science ( Bodó *et al.*, 2021). Though presented with some variation depending on context and discipline, decentralisation generally refers to the transfer of decision-making authority and resources from higher tiers or centralised structures to those in lower tiers, local and/or peripheries. This process aims to bring decision-making power closer to the people, enhancing the collective's efficiency, accountability, transparency and representativeness. The Organisation for Economic Co-operation and Development ( OECD, 2019) lists decentralisation among the most significant reforms of the past 50 years globally, with some form of decentralisation being implemented in varying degrees across the majority of both developed and developing countries. With regard to Africa and studies of Africa, the concept has been widely discussed and implemented across the continent, particularly since the late 20th century. African governments often inherited centralised governance structures designed for colonial administration, which were ill-suited for fostering inclusive development or local autonomy and decentralisation policies emerged as a way to devolve power, improve governance, and address the growing demand for citizen participation in decision-making ( Makara, 2018). In the 1980s and 1990s, global development partners, including the World Bank and the International Monetary Fund (IMF), strongly advocated for decentralisation as part of structural adjustment programs, linking it to good governance and poverty reduction ( Olowu, 2001; Rondinelli *et al.*, 1983). As such, decentralisation is widely recognised and embedded in public discourse globally, making it a good term to use as an analogy.

The likeness between decentralisation and decolonisation is easily recognisable. Decentralisation, broadly understood, involves re-distributing decision-making authority from centralised structures to localised actors, enhancing their agency and responsiveness. Similarly, decolonisation demands the re-attribution of power held and value accorded previously to Western thought and thinkers for liberation from Eurocentric epistemic oppression. Just as decentralisation remains a popular idea in Africa due to its promise of empowering communities, fostering participatory governance, and addressing local needs, decolonising knowledge production offers to enhance Indigenous peoples' capacity to determine what is known, how it is known, and their value as knowledge producers. Decentralisation involves re-distributing power and agency, not simply adding diverse perspectives to an unchanged system. So, by framing decolonising knowledge production as the decentralisation of epistemic power, we can better articulate that it requires a structural shift in how knowledge is valued and who is empowered to produce it. Just as governance decentralisation seeks to empower local actors and improve responsiveness, decolonising knowledge production (decentralisation of epistemic power) aims to empower historically marginalised groups to determine what kind of research is done, how it is done, how the knowledge is shared, and more- improving their engagement through more equitable participation.

Decolonising knowledge production seeks to dismantle epistemological hierarchies that privilege Western or dominant paradigms of knowledge. Framing it as the decentralisation of epistemic power foregrounds the need to recognise that knowledge is power and that coloniality and epistemicide robbed and continue to restrain the epistemic power of Indigenous and historically marginalised people. Thus, not only must the previously invalidated ways of knowing the subjects of the knowledge be resurrected, validated and integrated, but there is also a need to restructure epistemic infrastructures (academic traditions, institutions, and more) to ensure more contextually relevant decision-making and empower those who have been historically excluded from or marginalised within many parts of the knowledge production process.

The processes of decentralising and decolonising clearly share fundamental similarities rooted in the principles of equity, inclusivity, and the redistribution of authority. Both concepts challenge hierarchical systems that centralise control in the hands of a privileged few, advocating instead for more participatory and pluralistic approaches that value diverse contributions and perspectives ( Ndlovu-Gatsheni, 2021). As decolonising knowledge advocates for participatory research methodologies that position communities as co-creators of knowledge rather than passive subjects of study, so too decentralisation fosters participatory governance models, such as local councils or community-led development initiatives, where people have a direct say in the decisions affecting their lives. In both frameworks, re-distributing authority enhances agency, representation, and inclusion.

Both processes challenge the legacies of colonialism, imperialism, and oppression. Decolonising knowledge critiques the colonial roots of many academic and institutional systems that have imposed singular narratives while erasing or marginalising alternative voices. Decentralisation, on its part, critiques the centralisation of power in governance systems—often a colonial legacy—that perpetuates inequities and inhibits local agency ( Makara, 2018). In both cases, the aim is to restore balance and create systems that are more representative of the lived realities and aspirations of diverse populations, arguably demonstrating what has been termed as epistemic repair, attempts at epistemic reparations (see Horsthemke, 2025; Sriprakash, 2023; Walker, 2024).

Finally, both processes are iterative and transformative rather than transactional or finite. Decentralisation of power is not merely the delegation of authority but a shift in the underlying power structures to foster sustainable, long-term equity and resilience. Decolonising knowledge production also requires continuous interrogation of power dynamics within and between institutions of knowledge production, research processes, and global knowledge systems at large. Both decentralising and decolonising efforts envision systems where multiple voices are heard, respected, and integrated into decision-making processes, ensuring that no single perspective dominates.

***What this framing offers us.*** The primary advantage of framing the decolonisation of knowledge production as the decentralisation of epistemic power is that it provides a unified perspective on decolonial thought, addressing a significant challenge to the movement's progress. Yet, this framing equally makes decolonisation more actionable by directly tackling key issues within the discourse that have been previously highlighted in this paper.

For one, framing decolonisation as the decentralisation of epistemic power frees those who would pursue it from the false choice between those who call for radical rupture and and those who promote reformist efforts where absolute separation from colonial structures may be unrealistic. This framing enables the valuing of both revolutionary and incremental approaches by prioritising power redistribution as the core marker of decolonisation rather than focusing solely on its form or extent. Decentralisation efforts that efforts exist along a spectrum, and recognising decolonising as the decentralisation of epistemic power ensures that all actions—from renaming buildings to restructuring funding systems—contribute to shifting epistemic authority. Instead of debating whether an initiative is "decolonial enough," this framing emphasises meaningful steps toward re-distributing knowledge production power.

This framing also addresses the criticisms over the rigid dichotomy decolonising scholarship creates between 'Western' and 'African' knowledge systems and the common misconception that decolonisation simply involves replacing Western knowledge with indigenous alternatives. Such oversimplifications reduce decolonisation to a reactionary struggle rather than a call to move from epistemic hegemony towards epistemic plurality ( Ndlovu-Gatsheni, 2021). Using a decentralisation framing ensures that we do not create new hierarchies and hegemonies by substituting one epistemic framework for another but rather foster the coexistence of multiple knowledge traditions. This prevents the romanticisation of indigenous knowledge as an inherently pure alternative while ensuring its rightful place within a pluralistic system. By focusing on re-distributing authority within and between knowledge systems, this approach moves beyond merely identifying Eurocentric dominance and critiquing its harm to focus on how power operates within knowledge production. It also acknowledges that Africans, like others, may unintentionally perpetuate internalised colonial structures. In this way, decentralisation ensures that decolonisation is about equity in knowledge production, not replacing one epistemic authority with another. Decolonisation, then, is not just about rejecting Western epistemological superiority but also about dismantling the system that has foisted a rigid, one-size-fits-all model of knowledge production currently upheld by neoliberal globalisation and its push for "global universities" ( Ndlovu-Gatsheni, 2021). As Mbembe (cited in Ndlovu-Gatsheni, 2021 p.89) argues, decolonisation means bringing knowledge "as equitably as possible to everybody, every person and every text, every archive and every memory in the sphere of care and concern." This framing nurtures multi-vocal scholarship, ensuring diverse epistemologies engage in meaningful dialogue and that knowledge production remains proximate to the communities it concerns.

This approach highlights that all knowledge producers—even those from marginalised communities—hold power that must be equitably shared with research subjects and local communities. As a result, this framing applies across multiple levels. By Framing decolonisation as epistemic decentralisation, individual researchers must critically examine their positionality, not to absolve themselves ( Gani & Khan, 2024) but to decentre themselves and share their epistemic power and assess their role in furthering coloniality or epistemic hegemony through their selected methodologies, subjects, or how the knowledge they produce is disseminated. Likewise, this approach ensures institutions must reassess power asymmetries not only between Global North and Global South partnerships but even within South-South collaborations and within the African universities themselves to ensure they do not replicate the hierarchies they seek to dismantle ( Oelofsen, 2024). With the framing of decentralisation, the process of countering hegemony is more evident at individual, institutional, and regional scales.

With this framing of decolonisation as decentralisation and the focus on shifting epistemic power, we disrupt dominant narratives and reframe decolonisation as a global imperative rather than a 'favour' to the Global South. The need and benefits of decolonising knowledge are not limited to the Global South. Even knowledge created in and about the Global North is constrained by coloniality to restrictive canonised forms of intellectualising, limiting the possibilities for new ideas. Just as centralisation places pressure on one focal point, coloniality confines the Global North within rigid, traditional knowledge systems; therefore, decolonising knowledge via the decentralising of epistemic power offers the Global North liberation in a different way from how it offers the historically oppressed by providing fresh perspectives drawing from indigenous and alternative ways of understanding that could also address challenges in the West. By focusing on the re-distributing epistemic power of in and through knowledge production, the conversation on decolonisation is better presented as a necessary global effort to create a fairer, more open intellectual space. Changing the global knowledge system requires more than just rethinking how we study Africa—it calls for re-examining how knowledge is created everywhere and ensuring that no single perspective holds all the power.

A major critique of decolonisation discourse is its tendency to remain theoretical, with little tangible structural change emanating from it. Framing decolonisation as the decentralisation of epistemic power makes it more actionable by demanding scrutiny of how power is re-distributed and prioritising locally-led knowledge production, ownership, and dissemination. This ensures decolonisation is not merely an intellectual exercise but can be materialised with practical implications. As existing scholarship and frameworks establish, power disparities in knowledge production are evident across various dimensions of knowledge production, ranging from theoretical framing to resourcing, from methodological choices to language of dissemination ( AAU, 2023; Crawford *et al.*, 2021). Yet the decolonising rhetoric at this time often suggests that these imbalances are simply between Global South scholars and their Global North counterparts and, therefore, do little to illustrate how these imbalances can be addressed when they occur between African researchers and their less-privileged subjects or are replicated within and between Global South institutions as they pursue internationalisation. By framing decolonisation as re-distributing epistemic power, decolonising knowledge production becomes as practical and widely understood as decentralisation efforts in other domains. This framing provides clarity as to pathways on the spectrum of decolonising efforts and how one can move forward from decolonising discourse to action by addressing the deeply embedded power imbalances in knowledge production—whether at institutional or individual levels, in Global North-South partnerships, South-South collaborations, or within a young scholar's research.

Ultimately, regarding decolonisation as epistemic decentralisation simplifies the understanding of decolonising as efforts of re-distributing epistemic authority equity, and this makes pathways one can take in their pursuit of decolonising knowledge production all the more apparent. 

## Pathways for pragmatic decolonising of research

Thus far in this paper, I have argued that the decolonisation of knowledge is best understood through the lens of epistemic decentralisation. Framing decolonisation in this way not only makes the concept more accessible and actionable but also addresses critical challenges facing the decolonising movement today. By shifting the focus to how epistemic power is concentrated and controlled, this framework offers a clearer understanding of the mechanisms that sustain coloniality in knowledge production and highlights the pathways through which it can be effectively disrupted.

When viewed through this framing and considering my own experiences with decolonising research and the work of others I have engaged with; I find that the practical decolonisation of knowledge production tends to unfold through three general pathways of decentralising epistemic power. While somewhat distinct, these pathways are interconnected and represent the primary ways epistemic power is re-distributed. The first pathway I identify involves efforts to decentre dominant theoretical knowledge; here, dominant theoretical frameworks, ontologies, and paradigms are questioned, challenged, or replaced with alternative epistemologies that reflect historically marginalised perspectives towards an ecology of knowledges ( Martinez-Vargas, 2020). The second pathway encompasses efforts for the redistribution of methodological power and re-centring previously displaced knowledge producers; it focuses on decision-making around how, for whom and by whom knowledge is created, the methods used, the languages in which it is produced, and whose voices and perspectives are prioritised in the research process. The third and final pathway I identify entails decentring administrative epistemic power for systemic transformation; efforts here address the structural and institutional mechanisms that govern knowledge production, including funding allocation, knowledge dissemination, and the recognition of scholarship from historically marginalised regions and peoples.

In this latter part of the paper, I argue that all efforts toward decolonisation, regardless of their specific focus or context, ultimately involve decentralising one or more of these three dimensions of epistemic power. Drawing on my previous and ongoing research as well as examples from African scholarship, I outline the pathways identified to demonstrate for novice researchers and scholars across diverse fields how thinking of decolonising as decentralising of epistemic power would enable them to decide how best to go about their pursuit of decolonial knowledge production.

### Of past and previous research experiences

After my initial encounter with scholarship on decolonising knowledge production during my master's, I would make my first attempt at decolonising empirical research with my doctoral studies at the University of the Free State, South Africa (2018–2020). My research, entitled *Cameroonian Women's Empowerment through Higher Education: An African-Feminist and Capability Approach*, employed decolonial theoretical framing and methodology to challenge the assumption that higher education inherently empowers Cameroonian women—a belief that fuels societal fears of "overeducated" women threatening patriarchal norms. Noting the inadequacy of dominant Western feminist and human development frameworks to interrogate this context-dependent concept of empowerment sufficiently, I developed an African-feminist adaptation of the Capability Approach (AfCA) to examine what empowerment means in Cameroonian contexts and whether universities foster conditions for women to achieve their valued forms of empowerment.

The study engaged twenty female graduate students from four universities through participatory-narrative inquiry and collected data using life-story interviews and a co-created participatory analysis workshop. The combination of the AfCA and this methodology disrupted research hierarchies by positioning the women of whom empowerment is assumed, who I was to investigate, as knowledge co-creators rather than subjects. Participants interpreted their own narratives, bringing their perspectives directly into the analytical process. Ultimately, this decolonial approach demonstrated its unique utility by capturing the complex, intersecting nature of Cameroonian women's (dis)empowerment, which existing frameworks fell short of doing. Moreover, it enabled adequate challenging of universalist assumptions about education's transformative potential and underscored what sort of education would be needed for meaningful empowerment for Cameroonian women.

A few years later, my current role as a Cameroonian Research Associate on the earlier mentioned ERC-funded *Creative Lives of African Universities* (AFRIUNI) project has given me yet another opportunity to attempt decolonising empirical research. The project examines cultural and knowledge production, lived experiences, and representations of university life across four African countries—Senegal, Côte d'Ivoire, Benin, and Cameroon. In researching the life and lives of the University of Yaounde I, the Cameroonian case study, I apply a similar yet more elaborate strategy to my doctoral research. First, I engage in more theoretical decentering as I develop more contextually suited conceptual frames for analysis from adaptations of mainstream theoretical frameworks like cultural reproduction, social representation, multiculturalism, spatial (un)belonging and educational aspirations to African contexts by informing them with varied African epistemology. Next, as opposed to merely utilising a participatory (analysis) method, a key strand of the AFRIUNI project is designed as community-based participatory action research and engages 15 students (members of the university community and subjects of the research) from across the Faculty of Arts, Humanities and Social Sciences as co-researchers involved in decision-making at all stages of the research from conceptualisation to implementation and dissemination. The students collaboratively developed a 12-week PAR course through which we investigated the university's history, evaluated student experiences, aspirations, quality of education offered and systemic challenges encountered at the University of Yaounde I, ultimately presenting findings in both art and academic format. This research is still ongoing, and findings are not yet consolidated; thus far, the research has produced multimodal outputs- from audiovisual to demonstrating a plurality in epistemic expression in line with the decolonial objective of the ecology of knowledges ( de Sousa Santos, 2015; Ndlovu-Gatsheni, 2018). Efforts to decentralise epistemic power here also gave way to the student co-researchers' creation of products for their income generation and campus representation.

Through my first empirical attempt at decolonising research and my ongoing study, I have been able to experience all three pathways of epistemic decentralising – ergo, decolonising, which I identify here. However, beyond my own work, numerous examples from other scholars and declarations illustrate how decolonising research builds from the reflective hub to take on either or all of the pathways I identify here. Using examples of scholarship from scholars like Gabo Ntseane (2011) and Wunpini Mohammed (2022) and frameworks like the Bidirectional Emic-Etic (BEE) tool presented by Chilisa (2012) or the principles outlined in the African Charter, I outline in the subsequent subsections just how decolonising efforts emanate from the starting point (reflective hub) along these three pathways for decentralising epistemic power and decolonising knowledge production.

### Starting point: interrogation of self and purpose

As I considered the ways by which decolonising knowledge production efforts unfold, if and how researchers and institutions decide to decentralise power, I realised that beginning at the heart of all these efforts must be the recognition of (and commitment to re-distributing) the power dynamics and the inequalities embedded in knowledge production. Without that elementary point of deep introspection, even well-meaning initiatives risk reinforcing rather than dismantling colonial structures. Just as political decentralisation can fail without genuine transformation, epistemic decentralisation requires deliberate and critical engagement to avoid merely fragmenting coloniality instead of challenging it. Researchers must critically examine their positionality, motivations, and the power structures shaping their work. As Tuck and Yang (2012) argue, coloniality is deeply embedded in knowledge systems, making it easy for researchers to reproduce its logic even as they seek to resist it. Chilisa (2012) and Ahenakew (2016) also warn against the superficial integration of Indigenous epistemologies into dominant paradigms, which can subordinate rather than liberate them. Without careful reflection, decolonising efforts risk assimilation rather than genuine epistemic transformation. Yet, decolonising is an iterative process requiring ongoing critical reflection on varied power dynamics, context-specific needs, and structural inequities throughout the process of knowledge production. As such, this starting point is not a one-off stage to be ticked as suggested by most presentations of methodology chapters with positionality and ethics statements ( Gani & Khan, 2024), but a foundational hub to which the researcher [and institutions] must return again and again with every decision made to re-affirm their commitment to decolonising and ensure their work remains grounded in decolonial thought. It is necessary to note that this 'starting point' is not theoretical nor abstract. It is an ethical, ontological, and political act requiring a conscious commitment to dismantling epistemic oppression and its historical foundations. It is about repositioning marginalised peoples as knowledge creators and rethinking the very purposes, methodologies, and beneficiaries of research. The action requires answering fundamental questions: *What is being decolonised? From what? For whom? And to what end?* Framing decolonisation as epistemic decentralisation clarifies these concerns: *Who holds epistemic power? What knowledge has been canonised, what framing or method is dominant and whose perspectives have been excluded? Who determines validity in knowledge production?*

A crucial part of this reflection is recognising the deliberate displacement of Indigenous knowledge systems by colonial powers ( Ndlovu-Gatsheni, 2017). This is imperative because even where the decolonising effort looks like adapting existing methodologies, the adaptation must address epistemic theft, erasure, and systemic marginalisation as opposed to a 'mix and stir' format ( Ahenakew, 2016). The goal is not simply inclusion within dominant structures but the restructuring of those structures to restore and compensate for erased and undervalued knowledge. Addressing epistemic imbalances throughout the knowledge production process requires acknowledging not only the historical erasure of indigenous epistemologies and identifying displaced knowledge but also interrogating and dominant theoretical, methodological and structural practices for how they wither further, sustain or address coloniality and the hegemony of a certain type of knowledge and a select group of knowledge producers. Chilisa ( 2012 P.28–29) establishes the import of this starting point, where she sets out a schema for assessing decolonising intent, with key questions such as:

- *Does the research have social relevance, and is it transformative and participatory?*
- *Is the intent of decolonisation and indigenisation here explicit?*
- *Does the research take a stance against the political, academic, and methodological imperialism of its time?*
- *Does the research highlight potential areas of Western research incompatibility with local and indigenous epistemologies as well as areas of convergence?*
- *Does the research contribute toward the documentation and restoration of historically marginalised indigenous knowledge, cultures, and values?*

In my current work on the AFRIUNI project, this reflective hub has looked like regular evaluation/feedback exercises with co-researchers. On one of those check-ins, I was compelled to interrogate the extent to which the student co-researchers could be deemed co-researchers if they didn't have a research budget themselves. This eventually led to us engaging in participatory budgeting to organise the research restitution event following the PAR course. With every return to the starting point, this reflective process ensures that decolonising research is not a superficial exercise but a well-thought-out commitment to reconfiguring power dynamics in knowledge production.

### Pathway 1: challenging dominant epistemologies

The first, and I would argue, the most common pathway for decolonising knowledge production encompasses efforts towards decentralising conceptual and theoretical epistemic power; that is, the epistemic power to frame what is known and how it is known and establish what stands dominant as knowledge. Theoretical framing is fundamental to research, shaping how data is interpreted, concepts are defined, and conclusions are drawn. Decolonising research requires acknowledging how dominant discourses influence what is studied, what is valued, and ultimately who. For instance, colonial narratives frequently portray imperialism as beneficial to Indigenous populations, reinforcing the idea that imperialism 'gifted' the colonised with 'civilisation'; this framing is upheld by Eurocentric definitions of 'civilisation'. When a different conceptualisation of 'civilisation' is put forth, this idea is challenged, and colonialism can no longer be masked as 'beneficial'. Similarly, the framing of decolonising as 'removing' Eurocentricism from studies of and for Africans would suggest that only studies of and for formerly colonised people need to be decolonised. This would be incorrect as decolonising is not something to be done 'for' the colonised alone, but to ensure even the knowledge of the coloniser is more balanced.

Pathway 1 efforts generally challenge and decentralise dominant theoretical knowledge, exposing the ways in which dominant groups shape what is deemed valid knowledge, often distorting realities and countering distortions by highlighting alternative, contextually relevant epistemologies. Pathway 1 efforts towards decolonising are usually the entry point for decolonising. When asked how they intend to "decolonise their research," many novice researchers cite the use of Indigenous epistemologies. These efforts often involve critiquing Eurocentric perspectives, exposing their inadequacies, and either unearthing overlooked and previously dismissed indigenous epistemologies or developing alternative theoretical frameworks that better suit the specific contexts and issues.

 My earlier mentioned doctoral research exemplifies this approach. In investigating whether, how and to what extent higher education could be credited for empowering Cameroonian women, I recognised that 'best' evaluations of empowerment thus used the Human Development/Capability Approach typically informed by Western feminism. However, I recognised the inadequacy of mainstream feminist theories to capture the complexities of African women's realities (see Kwachou, 2020) and illustrated a Pathway 1 effort of decolonising as I developed an original African-feminist Capability Approach, which enabled me operationalised more suitable African Indigenous knowledge for investigation of African women issues.

Pathway 1 efforts (of all the paths) are likely more accessible to scholars from various fields and those outside of academia as well because questioning knowledge sources whose perspectives shaped the dominant beliefs/ideas and whether they are relevant to the context is something necessary for decolonial effort irrespective of field of study or work. For example, the BMI (Body Mass Index) as a framework for evaluating obesity has been challenged on the grounds that it was developed solely based on Eurocentric anatomical data and, therefore, is an unsuitable health metric for African and other indigenous populations. A reframing of health indicators integrated with indigenous solutions as put forward by public health scholars (see Warbrick *et al.*, 2019) is an example of Pathway 1 effort. Examples of Pathway 1 decolonising efforts are evident across both theoretical and empirical research. Motswana feminist-educationalist Gabo Ntseane, for instance, critiques Mezirow's transformative learning theory for its Western individualism. She integrates African values—such as cultural specificity, spirituality, community participation, and gender complementarity—to develop a more relevant framework. Similarly, Wunpini Mohammed (2022) applies the Dagbaŋ philosophy of *Bilchiinsi* to research methodologies in Northern Ghana, affirming the legitimacy of Indigenous knowledge systems.

Pathway 1 efforts tend to address power imbalances across three layers identified by the African Charter ( AAU, 2023 p.6); that is, imbalances in valuing of epistemologies (layer 1), theories and concepts in the generation of knowledge (layer 3), and the development frame (layer 4). While Pathway 1 is crucial for challenging dominant narratives and proposing alternatives, it does not always address the deep-seated structural changes needed for effective decolonisation. Where adaptation or counter-narratives are insufficient, more radical transformations become necessary, extending beyond this pathway's scope.

### Pathway 2: Re-centering displaced knowledge producers

Beyond the epistemic power of theory and conceptual framing, another critical imbalance in knowledge production lies in who is recognised as a knowledge producer, how knowledge is produced, and the limitations imposed on its presentation. Traditional research often positions scholars as authorities while relegating participants to passive subjects. Pathway 2 seeks to reverse this dynamic by ensuring those being studied actively shape research processes.

This pathway moves beyond challenging theoretical frameworks (as in Pathway 1) to directly addressing methodological power imbalances. It dismantles the hierarchy between the "knower" and the "known," a central critique of Eurocentric research traditions. Decolonial scholars argue that equitable knowledge production requires collaborative methodologies where participants influence the research purpose, shape data collection, set ethical boundaries, and co-interpret findings ( Chilisa, 2012). Rather than treating research as something done to or for communities, Pathway 2 reframes it as a co-creation of knowledge with them, decentralising the researcher where possible, empowering the subjects and democratising the process of the research as much as possible ( Martinez-Vargas, 2020).

Aside from researcher reflexivity (returning to the starting point), a key step in this process is the constant clear communication with the subjects of the research so problems are not assumed of them and decisions are not made for them without them. Pathway 2 efforts equally mean considering whom the research may leave out, who may be better positioned to lead the research (and empowering them to do so), how the sample is selected, and whether the case study choices and more may contribute to sustaining certain dominant perspectives. Participatory Action Research (PAR) is a typical attempt at decolonising research, which I would classify under Pathway 2 as it demands that communities be directly engaged in all research stages.

My own doctoral study employed the PAR method of participatory analysis for engaging Cameroonian women—graduate students presumed to be empowered by higher education—in a participatory analysis of their life narratives. While I initially selected data collection methods, I collaborated with participants to refine them. Instead of imposing a framework, I asked: How would you investigate the assumption that higher education empowers women like you? Their insights shaped a collaborative workbook used for assessing empowerment. Moreover, I openly shared African feminist theory and the Capability Approach, allowing participants to engage in their own interpretation of findings. This approach disrupted traditional hierarchies of meaning-making, mitigating epistemic injustices inherent in "Ivory Tower" research.

Similarly, in my current work with the AFRIUNI Project, I integrate participatory methods on a larger scale. The project prioritises current students as co-researchers, and they were engaged in co-designing a participatory research course—developing its curriculum, shaping pedagogical decisions, and co-creating knowledge through public art and discourse and then collaboratively managing a budget and production of the art-history exhibition where they showcased their outputs. Participatory approaches often invite subjects to be collaborators without evening out the playing field of sharing the researcher's knowledge or sufficiently equipping the participants so they can be equal co-researchers. With this project, funding enabled us to provide student co-researchers with the technology and information necessary to actively partake in the investigation and evaluation of the Cameroonian case study (see similar work showcased in Timmis *et al.*, 2024). This is how Pathway 2 empowers participants, not only inviting them to contribute but decentering the researchers' power so that the participants are equally able to collaborate in the knowledge production process.

Pathway 2 is not limited to participatory methodology; it also involves addressing inequalities, historical disadvantages, and subordination in the empirical processes of knowledge production. This entails efforts that would decentre the researcher and empower local communities/research participants such that issues like special needs accommodations, local language considerations, appreciation and compensations for participant's time and effort, community benefit, respect for customs and space, and other ethical guidelines receive due regard. A notable example of Pathway 2 effort would be the establishment of local research councils, where Indigenous groups establish ethical standards, protect their interests, and ensure researchers respect local norms. These councils counteract extractive research practices by shifting power from external researchers to the communities being studied.

Additionally, this pathway's ways efforts tend to ensure procedural respect to decolonial theory commitments, offering approaches and schema to resist the blind borrowing of universalised, Eurocentric paradigms or the inadequate integration of Indigenous ways of knowing in the study of the lived realities of marginalised communities (see Chilisa & Mertens, 2021).

As with efforts at any pathway, decolonising efforts can also fall short in Pathway 2. Power imbalances can persist even in participatory research, amplifying some voices while silencing others, especially across intersecting identities like race, gender, class, disability, religion and other status signifiers. To address this, researchers must remain power-conscious and adaptable, ensuring inclusivity and equity. Ultimately, Pathway 2 transforms research into a collaborative process that centres displaced knowledge producers. Through participatory action research, local research councils, or inclusive methodologies, it challenges academic hierarchies and advances decolonised knowledge production. By prioritising community interests and diverse modes of empirical engagement, this pathway re-distributes epistemic power and fosters just, inclusive knowledge production.

### Pathway 3: decentering administrative epistemic power

Pathway 3's decolonisation efforts are arguably the hardest to encounter and achieve but simultaneously the most transformative. Unlike Pathways 1 and 2, which focus on theoretical and methodological shifts, Pathway 3 requires systemic changes in how research is governed, funded, and operationalised. It demands a redistribution of administrative epistemic power, ensuring that decision-making over funding, partnerships, publishing, data access and more moves closer to the communities and scholars most affected by research outcomes. While individual researchers can challenge theoretical and methodological biases on their own upon their own persuasion, altering the administrative structures of knowledge production requires institutional change, making concrete examples of Pathway 3 efforts harder to implement.

Historically, Global North institutions have dictated research agendas, established the standards for legitimate knowledge, and controlled the dissemination of research, often to the exclusion of Global South scholars. This imbalance is rooted in colonial legacies that continue to privilege Western institutions. Global South scholars face systemic barriers that hinder their ability to engage as equal partners in research. Access to funding remains one of the most significant obstacles, as the majority of international research grants are administered by Global North institutions, which determine priorities and methodologies. Publishing opportunities are similarly restricted, with top academic journals primarily based in the Global North often requiring knowledge production to abide by Western academic traditions and engage Eurocentric epistemologies ( AAU, 2023; Afolabi, 2020). Even physical access to international academic spaces is limited by visa restrictions, preventing many Global South scholars from presenting their research in Global North venues. Pathway 3 efforts acknowledge these systemic inequalities and directly target the administrative and structural mechanisms that sustain them.

Yet, efforts to decentralise research administration often require researchers to challenge their own institutions, demanding the relinquishment or equitable sharing of epistemic power. The AFRIUNI Project serves as an example of these efforts in action. Initially conceived by Professor Ruth Bush at the University of Bristol and funded by the European Research Council, the project risked reinforcing traditional dependency models in which Global South researchers served as peripheral data collectors under a Global North principal investigator. Recognising this power imbalance, Bush took deliberate steps to re-distribute authority and establish equitable research governance.

A key aspect of this decentralisation was hiring research associates from the studied communities in Benin, Cameroon, and Côte d'Ivoire rather than treating them as secondary contributors. In addition, the project established a local council of research collaborators, composed of professors from case study institutions, who were given oversight responsibilities and compensated for their time and expertise. This structure ensured that decision-making was not concentrated in a UK-based institution but embedded within the studied communities. Research associates were granted autonomy to shape research objectives within their respective contexts, ensuring that knowledge production was led by those with lived experience rather than dictated by a Global North agenda. However, achieving this level of decentralisation required institutional advocacy and persistence, as UK hiring and immigration policies are designed to favour Global North researchers. The barriers encountered in securing equal status for Global South scholars underscore the extent to which administrative structures actively resist re-distributing epistemic power.

The impact of decentralising authority within the AFRIUNI project has been significant. By distributing decision-making among a Beninois sociologist, a Cameroonian feminist-educationalist, an Ivorian comparative literature scholar, and the UK-based principal investigator specialising in Francophone African Cultural Studies and Translation Studies, the project embraced epistemic and linguistic plurality. Each research associate adapted shared objectives to their local contexts, ensuring that methodologies were not externally imposed but instead reflected the realities and priorities of the studied communities. This autonomy enriched the project's findings and demonstrated the value of re-centring Global South scholars as knowledge producers.

Beyond the structural autonomy granted to research associates, decentralising administrative power had a trickle-down effect on the research process. As the Cameroonian research associate, I had the authority to hire local research assistants, procure research equipment, and determine the most appropriate methods for knowledge dissemination within the university community. These decisions were made locally, shifting the dynamics of research governance and reducing dependency within North-South collaborations. By ensuring that research resources were controlled by those directly affected by the study, the project challenged traditional funding hierarchies and placed power in the hands of local scholars.

However, for Pathway 3 to have a lasting impact, efforts must extend beyond individual projects and push for systemic reforms in the global research ecosystem. Initiatives like AFRIUNI may set a precedent, but these precedents must be leveraged to advocate for broader structural change. Policies prioritising the inclusion and leadership of Global South scholars are essential to making such efforts towards epistemic justice and decolonising permanent.

Pathway 3 efforts often expand upon those seen in Pathways 1 and 2, extending individual challenges into systemic change. For example, earlier identified Pathway 2 efforts toward more equitable language practices could take the form of making AFRIUNI publications available in both English and French with additional dissemination in Indigenous languages, counter linguistic hegemony and ensuring that research remains accessible to the studied communities (see Bush, 2016; Bush, 2024). At a broader institutional level, Pathway 3 efforts would involve support for multilingual publishing, prioritising indigenous translation services, and creating local-language journals, thereby enhancing the visibility of Global South research and disrupting English dominance in academic publishing.

Similarly, Pathway 1's efforts to decentralise dominant knowledge systems might involve researchers making a practice of deliberately citing scholarship from the case study country ( Okech, 2020). Expanding on this, Pathway 3 would push for institutional policies requiring a minimum percentage of references from scholars from the communities being studied. Additionally, strengthening African-based journals and encouraging scholars to publish in them would challenge Western academic dominance by elevating Indigenous knowledge systems and normalising their inclusion in global discourse.

These examples illustrate that the three pathways are interconnected, non-hierarchical, and non-linear. Rather than progressing step-by-step, they should be pursued concurrently, with decolonial commitments expressed across multiple levels.

## Conclusion

This paper is written for young scholars like my AFRIUNI student co-researchers—those who, like I once did, grapple with navigating the decolonisation of research. It is also for those outside academia, who may find the discourse on decolonising knowledge production overly theoretical or intimidating. To them, I offer two key arguments: first, that decolonisation can be understood as the decentralisation of epistemic power, and second, that just as decentralisation operates through multiple pathways, so too does the decolonisation of knowledge production. By framing it this way, I provide concrete routes for making decolonisation both accessible and actionable.

I hope that the analogy and illustrations in this work help further the transformation of decolonisation from an abstract ideal into a tangible pursuit that moves beyond rhetorical critique toward meaningful reparation and the restructuring of the global knowledge order. May this work serve as a tool for considering the re-distributing of epistemic power and cultivating a pluriversal landscape where African epistemologies are recognised as dynamic, generative, and integral to the global knowledge economy.

## Ethics and consent

No ethics and consent were required.

## Data Availability

No data are associated with this article.
