# Supplementary material for: Pathways for pragmatic decolonisation in research
Source: Open Res Eur. 2025 Apr 17;5:112. [Version 1] doi: 10.12688/openreseurope.19943.1 (PMC12149805; doi:10.12688/openreseurope.19943.1)
Supplement: Peer review history

# Reviewer report: Reviewer response for version 1

**DOI:** 10.21956/openreseurope.21572.r53449

**Published:** 2025-06-09

**Authors:** ‪Faith Mkwananzi‬

**Affiliations:** 1 University of the Free State, Bloemfontein, South Africa

The paper is well-written, conceptually rigorous, well-structured, and presents original, convincing arguments. The analytical approach is sound and engages meaningfully with current decolonisation and knowledge production literature. It draws on seminal and contemporary scholarship by authors such as Frantz Fanon, Linda Smith, Walter Mignolo, Sabelo Ndlovu-Gatsheni, and Bagele Chilisa. The author is abreast of recent scholarship, for example, drawing on the recent work of Emnet Woldegiorgis to support the framing of decoloniality. The robust engagement with current decolonial debates offers timely insights into evolving discussions in the field. The paper also critically engages with debates around the conceptual dilution and co-optation of decolonial discourse, thereby strengthening its conceptual contribution. Adopting “decentralisation” as a metaphor and framework for rethinking decolonial approaches provides a fresh lens to engage ongoing epistemic and decolonial discussions.

 The work is theoretically and methodologically robust. Although primarily conceptual, the paper draws from the author's empirical experience in two projects (doctoral research in South Africa and the AFRIUNI project in Cameroon), which are clearly described and well aligned with the paper’s theoretical aims. The author demonstrates reflexivity in their exposure to decolonial thought and provides examples from their teaching experiences. While empirically limited in scope, the reflexive approach enriches the papers’ conceptual argument and enables contextually grounded claims consistent with decolonial methods. Although not designed as a conventional empirical study, the paper provides rich detail on methodological processes used in both research projects. Other researchers could reasonably adapt these approaches, drawing on the methodological transparency evident in the paper. Also, as the paper draws heavily on personal and professional reflections, it suggests ways researchers could use similar reflexive approaches such as autoethnography, in different contexts. Such approaches are consistent with decolonial commitments as they challenge hierarchical knowledge production by elevating lived experiences. As the paper is not based on traditional empirical study or a quantitative method, the absence of empirical evidence is expected. The bibliographic information for consulted sources are references, claims made can be verified, and source materials accessed.

 In sum, the paper’s attempt at theoretical synthesis identifies a genuine challenge in decolonial scholarship fragmentation and offers a potentially valuable framework. The writing demonstrates an in-depth understanding of the field while proposing a constructive way forward. The three pathways (decentring dominant theories, redistributing methodological power, and decentralising administrative structures) are well substantiated with examples from both the author’s work and other scholars. However, the paper would benefit from more concrete examples and a detailed analysis of how this unifying framework could function in practice across diverse contexts. While offering strong claims about the framework’s potential impact, the conclusion would be strengthened by additional evidence or reflection on its feasibility, given African countries' heterogeneous political and institutional landscapes.

If any, are all the source data and materials underlying the results available?

Yes

Is the work original in terms of material and argument?

Yes

Is the work clearly and cogently presented?

Yes

Is the argument persuasive and supported by evidence?

Yes

Does the research article contribute to the cultural, historical, social understanding of the field?

Yes

Does it sufficiently engage with relevant methodologies and secondary literature on the topic?

Yes

Reviewer Expertise:

higher education

I confirm that I have read this submission and believe that I have an appropriate level of expertise to confirm that it is of an acceptable scientific standard.

# Reviewer report: Reviewer response for version 1

**DOI:** 10.21956/openreseurope.21572.r53455

**Published:** 2025-04-28

**Authors:** Pitshou Moleka

**Affiliations:** 1 Eudoxia Research Centre, Guwahati, India

The article presents a strong theoretical foundation for epistemic decentralisation and the decolonisation of knowledge, making important contributions to the field of postcolonial studies and African intellectual thought. The arguments are well-structured, and the writing is clear and compelling. However, there are several areas where the article could be strengthened:

 1° Incorporating Pitshou Moleka's Mode 4 Knowledge Production Framework: The article should integrate Moleka's Mode 4 as a critical element of the discussion on decolonisation.

 This framework offers a culturally specific approach to knowledge production that would complement the article's theoretical arguments and provide a concrete model for African-centred methodologies.

 2° Expanding the Methodologies Section: The article could benefit from a deeper exploration of methodologies, especially those that are participatory, community-based, and rooted in African traditions. This would strengthen the paper's argument for the decolonisation of research practices.

 3° Cultural, Historical, and Social Context: The article would be enriched by a more in-depth discussion of the specific cultural, historical, and social factors that influence African knowledge systems. This would further ground the theoretical arguments in the lived experiences of African communities and strengthen the cultural relevance of the paper.

 Recommendation: Accept with Minor Revisions

 The article presents a valuable contribution to the discourse on epistemic decentralisation and the

 decolonisation of knowledge. With the

 integration of Moleka's Mode 4 framework, a more comprehensive discussion of methodologies, the article would be stronger.

If any, are all the source data and materials underlying the results available?

No

Is the work original in terms of material and argument?

Yes

Is the work clearly and cogently presented?

Yes

Is the argument persuasive and supported by evidence?

Partly

Does the research article contribute to the cultural, historical, social understanding of the field?

Yes

Does it sufficiently engage with relevant methodologies and secondary literature on the topic?

Partly

Reviewer Expertise:

Decolonial thought and epistemologies, sociology of knowledge and postcolonial studies, innovationology, noesology, theology, leadership, transdisciplinary research , Mode 4 knowledge production,

I confirm that I have read this submission and believe that I have an appropriate level of expertise to confirm that it is of an acceptable scientific standard.
